# Quantitative ultrasound radiomics in predicting recurrence for patients with node‐positive head‐neck squamous cell carcinoma treated with radical radiotherapy

**DOI:** 10.1002/cam4.3634

**Published:** 2020-12-13

**Authors:** Archya Dasgupta, Kashuf Fatima, Daniel DiCenzo, Divya Bhardwaj, Karina Quiaoit, Murtuza Saifuddin, Irene Karam, Ian Poon, Zain Husain, William T. Tran, Lakshmanan Sannachi, Gregory J. Czarnota

**Affiliations:** ^1^ Department of Radiation Oncology Sunnybrook Health Sciences Centre Toronto Canada; ^2^ Department of Radiation Oncology University of Toronto Toronto Canada; ^3^ Physical Sciences Sunnybrook Research Institute Toronto Canada; ^4^ Evaluative Clinical Sciences Sunnybrook Research Institute Toronto Canada; ^5^ Department of Medical Biophysics University of Toronto Toronto Canada

**Keywords:** head‐neck squamous cell carcinoma, machine learning, quantitative ultrasound, radiomics, radiotherapy, recurrence, texture analysis

## Abstract

This prospective study was conducted to investigate the role of quantitative ultrasound (QUS) radiomics in predicting recurrence for patients with node‐positive head‐neck squamous cell carcinoma (HNSCC) treated with radical radiotherapy (RT). The most prominent cervical lymph node (LN) was scanned with a clinical ultrasound device having central frequency of 6.5 MHz. Ultrasound radiofrequency data were processed to obtain 7 QUS parameters. Color‐coded parametric maps were generated based on individual QUS spectral features corresponding to each of the smaller units. A total of 31 (7 primary QUS and 24 texture) features were obtained before treatment. All patients were treated with radical RT and followed according to standard institutional practice. Recurrence (local, regional, or distant) served as an endpoint. Three different machine learning classifiers with a set of maximally three features were used for model development and tested with leave‐one‐out cross‐validation for nonrecurrence and recurrence groups. Fifty‐one patients were included, with a median follow up of 38 months (range 7–64 months). Recurrence was observed in 17 patients. The best results were obtained using a k‐nearest neighbor (KNN) classifier with a sensitivity, specificity, accuracy, and an area under curve of 76%, 71%, 75%, and 0.74, respectively. All the three features selected for the KNN model were texture features. The KNN‐model‐predicted 3‐year recurrence‐free survival was 81% and 40% in the predicted no‐recurrence and predicted‐recurrence groups, respectively. (*p* = 0.001). The pilot study demonstrates pretreatment QUS‐radiomics can predict the recurrence group with an accuracy of 75% in patients with node‐positive HNSCC.

Clinical trial registration: clinicaltrials.gov.in identifier NCT03908684.

## Graphical abstract

Quantitative ultrasound radiomics obtained before starting treatment was used to predict the outcomes in patients with head‐neck squamous cell carcinoma treated with radical radiotherapy. A KNN classifier was able to predict the recurrence with an accuracy of 75%.

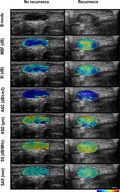

## 1 INTRODUCTION

Head and neck malignancies were the seventh most common cancer type globally in 2018, with 890,000 new cases diagnosed and responsible for 450,000 deaths.^1^, ^2^ In general, head and neck cancers account for diverse primary sites, including cancers of the thyroid, nasopharynx, larynx, oral cavity, oropharynx, salivary glands, and others. The majority of the malignancies arise from the epithelial lining of the anatomic subsites in the head‐neck region, with squamous cell carcinoma accounting for approximately 90%.^3^, ^4^ Head and neck squamous cell carcinomas (HNSCC) arising from the oropharynx, hypopharynx, larynx are often treated with organ preservation techniques with radical radiotherapy (RT) with or without concurrent chemotherapy (depending upon disease stage, age, medically fit patients).^2^ Given the anatomic and physiologic complexity of the head‐neck region, treatment‐related toxicities can have significant implications on the quality of life.^5^ Technological advances in the past decade in the form of intensity‐modulated radiotherapy (IMRT) and image guidance (IGRT) have helped in reducing the toxicities like xerostomia and dysphagia.^6^, ^7^ During presentation, approximately 40–60% of patients have locally advanced cancer, which constitutes advanced primary tumors with involvement of surrounding structures, and/or regional LN metastases, and are usually associated with a poor prognosis.^2^, ^4^

The clinical outcomes of HNSCC are driven by several factors like the site of primary disease, stage during diagnosis, as well as other factors (including molecular features like human papilloma virus, patient performance status, risk factors like tobacco use, etc.).^2^, ^4^ Imaging forms an integral part in the management of head‐neck malignancies, serving a crucial role in disease staging, treatment (e.g., radiation planning), the assessment of treatment response, and surveillance. Different imaging modalities are used in HNSCC, including ultrasonography (US), computed tomography (CT), magnetic resonance imaging (MRI), and positron emission tomography (PET). In recent years, the application of imaging has extended beyond the traditional role of diagnostics with the introduction of computational techniques. The field of “radiomics” involves advanced quantitative analysis of images facilitated by artificial intelligence to correlate imaging features with biological endpoints like molecular characteristics, perform risk‐stratification, and to predict treatment response and clinical outcomes.^8^, ^9^ The ability of noninvasive characterization with imaging has generated tremendous interest in the application of radiomics and to serve as useful biomarkers, a step forward toward precision oncology. A significant body of emerging research is available related to radiomics in HNSCC using different treatment modalities primarily using CT, MRI, or PET with encouraging results.^10^, ^11^, ^12^

There is limited data investigating the utility of US‐based radiomics in HNSCC. Ultrasound is a simple, inexpensive, easily accessible portable imaging modality with rapid scan acquisition and excellent patient compliance. B‐mode US has a well‐established role in HNSCC, particularly in the determination of LN metastasis and is often undertaken to guide and obtain tissue diagnosis in suspicious sub‐clinical neck nodes.^13^, ^14^ Quantitative ultrasound (QUS) is similar to the B‐mode US in terms of scan acquisition, with the advantage of processing raw radiofrequency (RF) data, which retains more detailed information characterizing what has been insonified.^15^ The elastic properties of the tissue at the microcellular level, as probed by ultrasound, serve as a surrogate of biological characteristics and can be further processed to generate useful information. The various spectral parameters reflect intrinsic tissue properties like cellular density, scatterer size, and tissue organization, which correlates with biological outcomes.^16^, ^17^, ^18^, ^19^, ^20^, ^21^ Texture analysis of the spectral features of the tissue can further help in characterizing the heterogeneity of the tumor, which often is linked to the responsiveness to treatment and clinical outcomes. An earlier report involving 32 patients, detailed that pretreatment QUS features could predict the response to RT in patients with head‐neck malignancies.^22^ The present study investigates the role of QUS‐radiomics obtained before starting RT in classifying recurrence groups for 51 patients with HNSCC. The study here represents the first clinical investigation on the use of QUS‐radiomics in recurrence‐risk estimation in patients with HNSCC treated with radical RT.

## 2 MATERIAL AND METHODS

### 2.1 Patient selection

The prospective observational study was undertaken at the Sunnybrook Health Sciences Centre, Toronto, approved by the institutional Research Ethics Committee. The trial was registered with clinicaltrials.gov.in (identifier NCT03908684). The study was conducted according to the declaration of Helsinki following good clinical practice and monitored by the institutional ethics committee. All patients were needed to have a diagnosis of biopsy‐proven HNSCC with primary site of oropharynx, hypopharynx, and larynx (inclusive of carcinoma of unknown primary with neck nodes) with clinically apparent neck nodes amenable to ultrasound imaging. Patients decided to be treated with radical intent RT (with or without concurrent chemotherapy), with no prior cancer‐directed therapies, were considered eligible for the study. Patients with evidence of distant metastasis were excluded from the study. A written consent form was obtained from all the patients. In patients with carcinoma unknown primary (CUP), the histology from LN showing features suggestive of nasopharyngeal carcinoma or expression of Epstein–Barr virus were excluded from the study. A minimum follow up of 12 months was considered for patients without any evidence of disease recurrence. Patients developing second primary in the head‐neck region or other areas (e.g., lung, esophagus) were excluded from the current study. The study accrual was done between January 2015 and June 2018. The survival data were locked for the final analysis in May 2020.

### 2.2 Treatment protocols

All the patients in the study were treated according to standard institutional protocols without any influence of the study. All the patients were treated with IMRT and IGRT techniques with a dose prescription of 70 Gy/33 fractions in 6–7 weeks to the high‐risk volume. The decision regarding concurrent systemic therapy was at the discretion of medical oncologists depending upon age, performance status, comorbidities following standard guidelines. Following treatment completion, the response was assessed at approximately 3 months with standard imaging (CT/ MRI) and clinical examination with endoscopy and functional imaging (PET) as decided by the treating oncologists. Patients with residual disease were closely followed up with additional investigations as indicated. Follow up was undertaken every 3–6 months in the initial 2 years and thereafter every 6–12 months. Disease recurrence was confirmed by the responsible clinicians with clinical examinations, imaging, and tissue diagnosis as appropriate. All the patients with suspected recurrent disease were discussed in a multidisciplinary tumor board constituting radiation oncologists, surgeons, medical oncologists, radiologists, and pathologists with expertise in head and neck malignancies. In general, for patients with local and nodal recurrence, endoscopic examination, cross‐sectional imaging, and biopsy were undertaken. Any patients with disease recurrence had undergone repeat staging investigations with CT neck, thorax, abdomen, pelvis with a bone scan or PET‐CT, and other symptom directed investigations (like MRI brain or spine). In general, for patients with disseminated metastatic disease, no additional histology was acquired, unless other differentials were considered (e.g., single lung lesion to rule out the second primary).

### 2.3 Quantitative ultrasound and image analysis

The most prominent LN amenable to imaging was decided by the research sonographer in conjunction with the radiation oncologist. As a part of the study protocol, the index LN was required to have a size of more than 1 cm. The LN selected was ideally needed to be over an area accessible to ultrasound imaging (e.g., retropharyngeal were excluded). In general, the largest LN (or conglomerate nodal mass) was selected for imaging. Although the target LNs were not subjected to histological confirmation (unless CUP), the scanned LNs were included only if they had a strong radiological suspicion (ultrasound, CT/MRI/PET) of metastatic involvement as determined by radiation oncologists and radiologists and underwent treatment for disease involvement. Lymph nodes were included irrespective of the presence of radiological extranodal extension. The QUS scan was obtained before the start of RT (preferably within 24 hours, an interval of 1 week was allowed). The ultrasound data were acquired using a clinical ultrasound system with standardized settings for ultrasound parameters (Elekta Ltd, Montreal, Canada) with a linear 4‐D transducer (4DL14‐5/39 Linear 4D, BK Ultrasound) or a Sonix RP clinical ultrasound system (Analogic Medical Corp.) with a linear array transducer (L14‐5/60). The center frequency was 6.5 MHz (bandwidth 3–8 MHz). The sampling frequency for both the device was 40 MHz, and the focal depth was 2.5 and 1.75 cm, respectively.

The target LN was manually contoured designated as the region of interest (ROI). For each ROI, 3–5 slices were obtained at regular intervals encompassing the entire LN. The RF data collected from the ROI were divided into smaller blocks using a sliding window technique with a 92% overlap along the axial and lateral directions, corresponding to linear dimensions of approximately 2 mm × 2 mm. A fast Fourier transformation was applied to the raw RF data from each unit to generate the power spectrum, which was normalized using a tissue‐mimicking phantom serving as a reference. Seven QUS spectral parameters were obtained from the RF data‐spectral slope (SS), spectral intercept (SI) at 0 MHz, mid‐band fit (MBF), average acoustic concentration (AAC), average scatterer diameter (ASD), attenuation coefficient estimate (ACE), and spacing among scatterers (SAS). The spectral parameters obtained from each of the smaller units from all the slices were averaged and served as first‐order features. The details of image processing, standardization of parameters, texture feature extraction had been described in previous publications.^18^, ^23^, ^24^

For texture analysis, the color‐coded QUS‐parametric maps were generated using the quantitative estimates from each of the smaller units for individual spectral parameters (except ACE). A grey level co‐occurrence matrix (GLCM) method was used to generate texture features to compute the relation with neighboring pixels (1,2,3,4) for angular relations of 0°, 45°, 90° and 135°. Four texture features were extracted‐energy (ENE), contrast (CON), homogeneity (HOM), and correlation (COR). Therefore, a total of 24 QUS‐texture features were obtained from six spectral features leading to a total of 31 features. We used the weighted average (depending upon the area of individual slices) for all the individual spectral and texture parameters to generate a single value for concerned parameters. The individual features extracted from all the patients were normalized using a “Z‐score normalization” technique.

### 2.4 Statistical analysis and machine learning classifiers

The endpoint (ground truth) for the study was recurrence and no‐recurrence as obtained from the clinical outcomes. A Shapiro–Wilk test was performed to study the distribution of the data between the two groups. Unpaired t‐tests were performed for normally distributed data, while Mann–Whitney tests were undertaken for nonparametric data. Three machine learning classifiers were used to develop the radiomics model‐Fisher's linear discriminant (FLD), k‐nearest neighbor (KNN), and support vector machine (SVM). The KNN classifier was used with different k values of 1, 2, 3, 4, and 5. For the SVM classifier, parameters C and γ were optimized using a grid search method (C and γ ranging from 2^0^ to 2^10^). A sequential forward feature selection method was used for data classification, using a maximum of three features to avoid overfitting of the model, given the smaller number of samples. The number of features was limited to 3, given the number of patients in the “no‐recurrence” group was 34, following the rule of thumb of using n/10 features for optimal classification. The feature selection method first analyses the best feature for classification and subsequently keeps adding the next set of features to the one already selected, to achieve the best classifier performance. As the number of subjects was unevenly distributed between the two groups, seven subsets were generated, selecting an equal proportion of patients from each of the groups in a random manner. The final classifier results were the values obtained from the combination of the individual subsets. The subset sampling involved downsampling the majority group (no‐recurrence) and training the algorithm. This process was run for seven iterations, with the final label (predicted recurrence vs. predicted no‐recurrence) decided through majority voting. Leave‐one‐out cross‐validation was performed to test the efficacy of the models and obtaining the confusion matrix. The method involves training the classifier algorithm with all subjects except one, which is used to test the algorithm. The process is repeated across the entire cohort until all the subjects are left out once. Receiver operating characteristics (ROC) was used to generate the area under curve (AUC) values. Kaplan–Meier product‐limit method was used for survival analysis. The date of the histopathological diagnosis of HNSCC was considered as the baseline date for survival analysis. The final influence of predicted groups from each of the classifiers (predicted recurrence vs. predicted no‐recurrence groups) on the recurrence‐free survival was tested using a log‐rank test. The segmentation, feature extraction, and machine learning classification were done using MATLAB (MathWorks Inc., USA). For statistical significance, a p‐value of <0.05 was considered significant.

## 3 RESULTS

### 3.1 Clinical characteristics

The analysis here included 51 patients with HNSCC. For the entire group, the median age was 60 years (range 39–80 years), with 10 (20%) patients aged 70 years or above. The different patient, disease, and treatment characteristics are summarized in Table 1. The most common site of primary disease was the oropharynx in 39 patients, followed by the larynx in 5, CUP in 5, and hypopharynx in 2. Of the 38 patients with known molecular disease status, 36 (95%) had positive p16 ‐immunostaining suggestive of human papilloma virus (HPV)‐related disease. Concurrent chemotherapy was administered in 41 patients, with the majority receiving cisplatinum (three started with cisplatinum and later switched to carboplatinum, and three received carboplatinum alone). Cetuximab was used concurrently with RT in one patient (no chemotherapy).

**TABLE 1:** Clinical characteristics for patients with recurrence and without disease recurrence.

Clinical features		Recurrence (n = 17)	No Recurrence (n = 34)
Patient characteristics			
Age	Median (Range)	59 (40–70) years	61 (39–80) years
Gender	Female	0	3
	Male	17	31
Smoking Status	Smoker	12	23
	Non‐Smoker	5	11
Disease Characteristics			
T‐stage	T0a	4	1
	T1	0	14
	T2	4	14
	T3	3	2
	T4	6	3
N‐stage	N1	1	21
	N2	8	12
	N3	8	1
Site	Oropharynx	10	29
	Hypopharynx	1	1
	Larynx	2	3
	CUP	4	1
HPV p16 stain	Positive	8	28
	Negative	2	0
	Indeterminate/Unknown	7	6
Treatment characteristics			
Concurrent chemotherapy	Cisplatin	10	25
	Cisplatin >Carboplatin	1	2
	Carboplatin	1	2
Concurrent biological therapy	Cetuximab	1	0
Radiation Alone	Radiation Only	4	5

Abbreviations: CUP, Carcinoma of unknown primary origin; HPV, Human Papilloma Virus.

^a^ Carcinoma Unknown Primary

### 3.2 Clinical outcomes

The median follow up for all patients in the study was 38 months (range 7 to 64 months). For patients without recurrence, the median follow up was 42 months (range 14 to 59 months). In the study here, 15 of 51 patients had a complete response in primary and lymph nodes at 3 months. Of the remaining 36 patients on further follow‐up, the disease had resolved in 28 patients, with a median time of 6 months (range 4–9 months). In all eight patients with residual disease, disease progression was seen at different times. The recurrence‐free survival (RFS) at 2 and 5‐year was 72% and 62%, respectively. A total of 17 patients had disease recurrences during the study period. The predominant site of relapse involved distant sites in 13 patients, regional nodes in 8, and local sites of primary disease in 4 (alone or in combination), as shown in Figure S1. The median time to disease recurrence (from diagnosis) was 9 months (range 1 to 48 months), with more than 80% recurrences encountered in the initial 2 years. The 2 and 5‐year overall survival (OS) for all patients was 90% and 64%, respectively.

### 3.3 Feature analysis and classifier results

**FIGURE 1:**
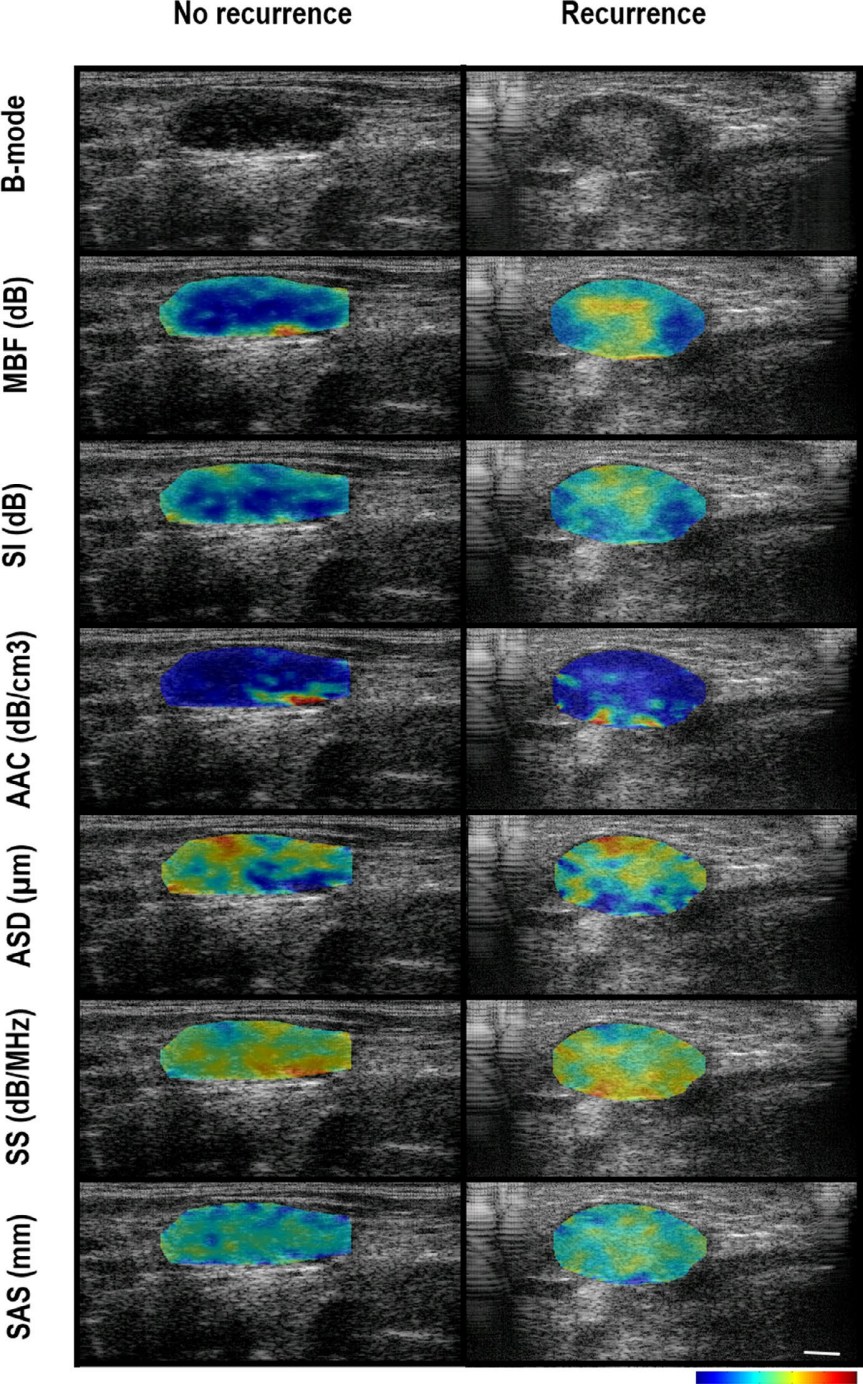
Representative ultrasound B‐mode images (upper row) with six spectral parametric maps from two patients—no recurrence (left panel) and recurrence (right panel). Parametric images from top to bottom represent overlays of the MBF, SI AAC, ASD, SS, and SAS parameters. The white scale bar (right lower corner) represents a length of 5 mm. The color bars present the range for MBF parameter of −10 dB to 25 dB, SI parameter of −10 dB to 60 dB, AAC parameter of 20 dB/cm‐MHz to 170 dB/cm‐MHz, ASD parameter of 1 µm to 200 µm, SS parameter of −8 dB/MHz to 22 dB/MHz, and SAS parameter of 0.2 mm to 2.5 mm

**FIGURE 2:**
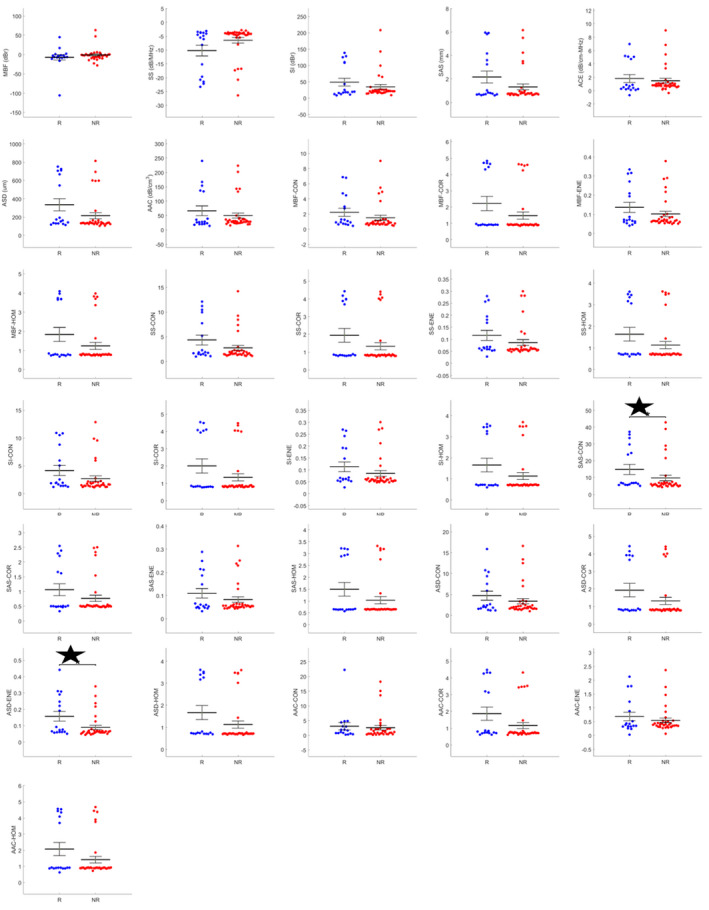
Scatter plot presenting the distribution of values for QUS features. Blue symbols represent patients with recurrence (R), while the red denotes the patients with nonrecurrence (NR). The two highlighted features (stars) are SAS‐CON and ASD‐ENE, which had a distribution between the two groups reaching statistical significance

Representative B‐mode and the QUS parametric maps are presented in Figure 1 for patients, one each from the two groups (recurrence and no‐recurrence). There was obvious intra‐tumoral heterogeneity in quantitative ultrasound parameters evident in the parametric images. Two texture features, SAS‐CON and ASD‐ENE, had significantly different distributions between the two outcome groups (Table S1) as single discriminatory parameters. The mean values for SAS‐CON and ASD‐ENE were higher in patients with disease recurrence compared to patients without recurrence with *p*‐values of 0.049 and 0.026, respectively. The scatter plots showing the distribution of all features between the two groups are presented in Figure 2. On their own, the majority of parameters were not statistically discriminant between the two groups.

**FIGURE 3:**
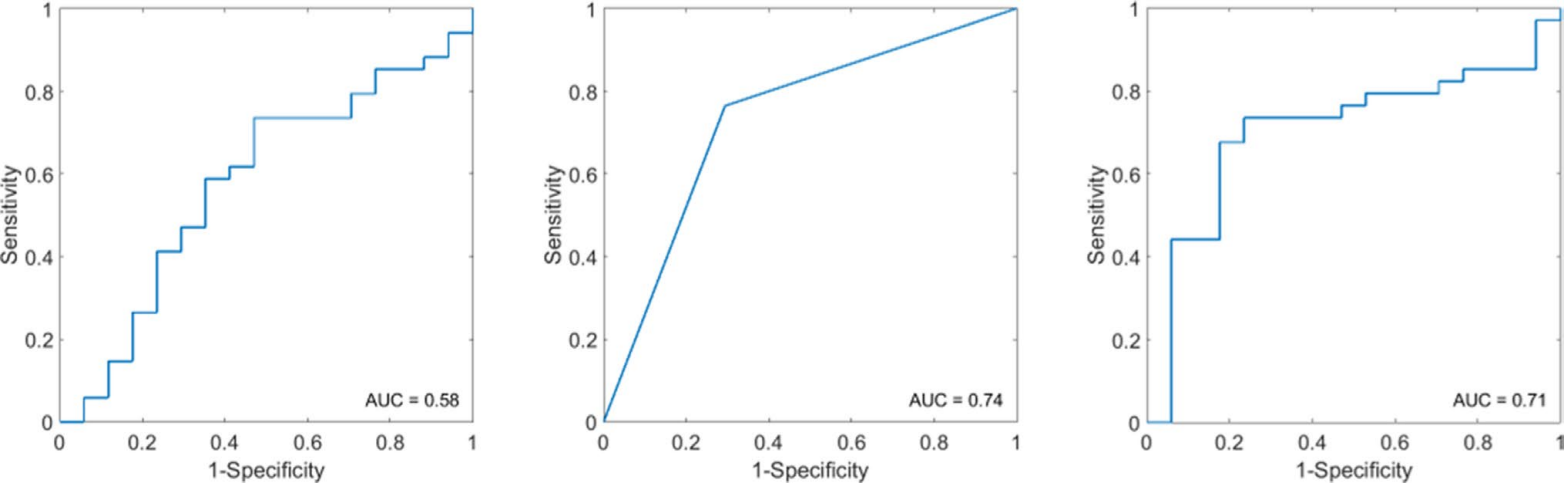
The receiver operating characteristics (ROC) curves for the three models using Fisher's linear discriminant (A), k‐nearest neighbor (B), and support vector machine (C) classifiers

Figure 3 represents the ROC curves using the three classifiers. The best classifier results were obtained using a KNN‐based model and demonstrated a sensitivity, specificity, accuracy, and AUC of 76%, 71%, 75%, and 0.74, respectively, for *a priori* to treatment predicting recurrence (Table 2). The three selected parameters in the KNN classifier were QUS‐texture features SS‐ENE, SI‐ENE, and MBF‐COR. The SVM‐model was slightly inferior to the KNN‐model in terms of accuracy and AUC, although the specificity of 75% was higher than the KNN‐model (71%). The SVM‐model selected two QUS spectral features (ACE and SI) and the third one being a texture feature (SI‐CON). The results from the FLD model was unsatisfactory for each of the indices compared to the other two models.

**TABLE 2:** The classification performance of the three machine learning models with the best features selected

Model	Sensitivity %	Specificity %	Accuracy %	AUC	Best feature(s)		
FLD	59	55	57	0.58	MBF^a^		
KNN	76	71	75	0.74	SS‐ENE	SI‐ENE	MBF‐COR
SVM	72	75	73	0.71	ACE	SI	SI‐CON

Abbreviations: ACE, Attenuation coefficient estimate; AUC, Area under curve; CON, Contrast; COR, Correlation; EE, Energy; FLD, Fisher's linear discriminant; KNN, k‐nearest neighbor; MBF, Mid‐band fit; SI, Spectral intercept; SS, Spectral slope; SVM, Support vector machine.

^a^ One feature was selected as further feature addition did not lead to improvement of the classifier performances.

**FIGURE 4:**
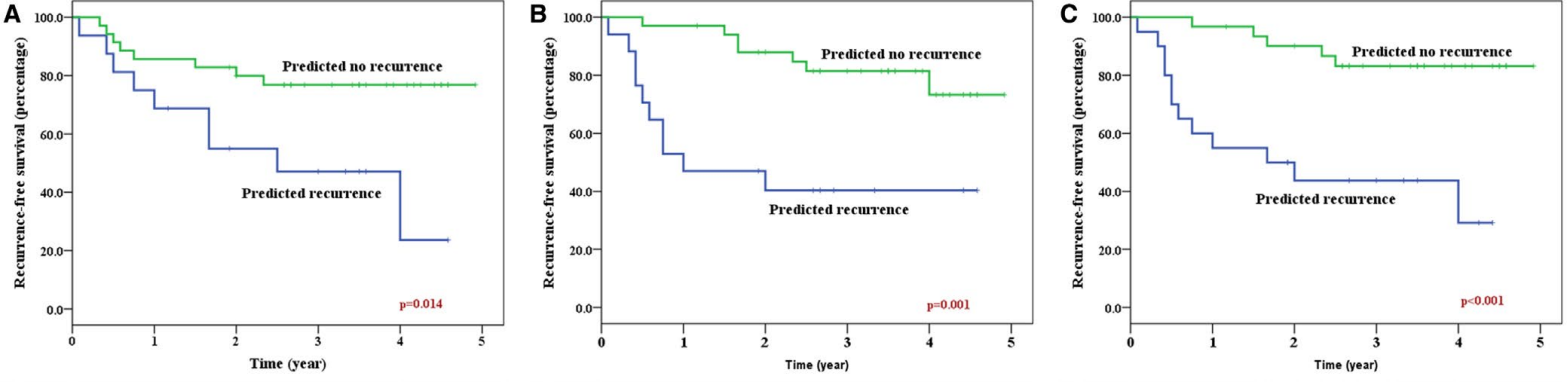
Kaplan–Meier survival plots showing the predicted recurrence‐free survival obtained using Fisher's linear discriminant (A), k‐nearest neighbor (B), and support vector machine (C) classifiers

The RFS, using the three model‐based predictions, has been shown in Figure 4. Using the KNN‐model, the 3‐year RFS for the predicted recurrence and nonrecurrence groups were 40% and 81%, respectively (*p* = 0.001). Similarly, for the SVM‐model, the predicted 3‐year RFS was 44% and 83% for the predicted recurrence and predicted nonrecurrence groups, respectively (*p* < 0.001).

## 4 DISCUSSION

Head and neck malignancies encompass a diverse group of cancers involving the head‐neck anatomical region, with the majority arising from the epithelial lining of the upper aerodigestive system or the various glandular structures (thyroid, salivary glands). For HNSCC, the outcomes vary widely, with excellent cure rates seen in very early‐stage cancers, while advanced and metastatic tumors are associated with guarded prognosis.^2^ For locally advanced HNSCC or node‐positive disease, 5‐year survival rates had been reported to be less than 50%.^2^ With the advent of the era of radiomics, there are promises in the development of effective risk‐stratification strategies using noninvasive imaging biomarkers, which can be potentially adopted in personalized medicine.^25^ The study here investigated the efficacy of pretreatment QUS‐radiomics in the prediction of recurrence for patients with node‐positive HNSCC treated with radical RT.

Several distinct risk factors (HPV, tobacco, alcohol, etc.) and molecular pathways (epidermal growth factor receptor, aberrant p53, etc.) have been identified in the etiopathogenesis of HNSCC.^2^, ^26^, ^27^, ^28^ Genetic markers and liquid biopsy using circulating tumor DNA had been investigated in prognostication and predicting recurrence in patients with HNSCC with variable degrees of success.^29^, ^30^, ^31^, ^32^ In recent years there has been an increasing interest in the use of imaging markers utilizing various imaging modalities that are part of standard treatment protocols. Also, radiomic analysis can be undertaken noninvasively during the treatment leading to an early characterization of treatment responses compared to traditional imaging assessment, which accounts for structural changes manifested after several months. As morphological (CT, MRI) or functional imaging (PET) is widely used in diagnostics, staging workup and RT planning for HNSCC, several groups have undertaken radiomics‐based approaches using different clinical endpoints.^10^, ^11^, ^12^ Pretreatment CT image‐based radiomics have been used in the identification of HNSCC associated with HPV and other molecular features.^33^, ^34^ In a recent multi‐institutional study, features of preoperative contrast‐enhanced CT images have been shown to correlate with the extranodal extension on histopathological examination using deep learning models.^35^ In a study involving 465 patients with oropharyngeal cancer (OPC), pretreatment contrast‐enhanced CT texture analysis could stratify patients into risk groups with different local control rates.^36^ Similarly, in a cohort of 300 patients with HPV‐related OPC, Kwan et al had demonstrated CT planning scans used for RT planning could be used to identify patients at a higher risk of distant metastasis.^37^ The most common application of MRI‐based radiomics in risk‐stratification had been undertaken in nasopharyngeal carcinoma.^38^ Yuan et al had indicated T2‐weighted MRI in patients with HNSCC (mostly oral cavity and OPC) can serve as an independent prognostic marker.^39^ Vallières et al had performed a radiomic analysis of pretreatment PET and CT images in 300 patients with head‐neck cancer, which was able to predict locoregional relapse and distant metastasis in independent cohorts with AUCs of 0.69 and 0.86, respectively.^40^

In the study here, best results were obtained using a KNN‐based QUS model with an accuracy of 75% and AUC of 0.74, which is comparable to several previously reported studies. QUS imaging has been widely studied in breast cancer and established its efficacy in demarcating between different tumor grades, response prediction and the monitoring of response during treatment.^23^, ^24^, ^41^ The use of QUS in head‐neck malignancies is a new application and has demonstrated an accuracy of 88% in predicting response to RT in 32 patients.^22^ QUS Imaging relies on microcellular tissue architecture, which is represented by the various spectral parameters determined by QUS.^18^ The KNN model selected texture features related to SS, SI, and MBF in classification between the two groups of patients (SS‐ENE, SI‐ENE, and MBF‐COR). The SS is determined by the scatterer size and shape, while the SI depends upon scatterer concentration, and the MBF is influenced by various elastic properties of the tissue. These findings suggest a differential tissue architecture for tumors with different biological behavior.

Texture features determined information related to tumor heterogeneity, which is known to influence the clinical outcomes. Intra‐tumoral heterogeneity was evident within different areas of the tumoral masses, which can lead to the development of treatment resistance and impact survival.^42^, ^43^ In general, tumor heterogeneity is associated with more aggressive tumor behavior, as also indicated here.

This is the first study demonstrating the potential of QUS‐based radiomics obtained *a priori* to treatment as an imaging modality in predicting recurrence (local, regional or distant) in patients with HNSCC. This can be adopted in clinical practice and utilized to take a step toward personalizing treatments with intensification strategies for higher risk patients predicted to have a recurrence. One of the limitations of the current report is a relatively small number of patients; however, the results point to being able to predict tumor behavior and its impact on survival using quantitative ultrasound methods. In the future, with the expansion of study cohorts, it should be possible to use advanced classifiers like deep learning and test the effectiveness of the model in independent groups to increase reliability and clinical utility.

## 5 CONCLUSION

The study presented here demonstrates the effectiveness of a pretreatment QUS‐radiomics model in predicting recurrence for patients with HNSCC treated with radical RT with reasonable accuracy. A KNN‐based model provided the best classifier results with an accuracy of 75% and an AUC of 0.74.

## CONFLICTS OF INTEREST

None to declare.

## AUTHOR CONTRIBUTIONS

**Archya Dasgupta**: Study concept, investigation, resources, formal analysis, methodology, writing‐original draft, review, editing. **Kashuf Fatima**: Investigation, resources, formal analysis, methodology, writing‐original draft, review, editing. **Daniel DiCenzo**: Investigation, resources, formal analysis, methodology, writing‐original draft, review, editing. **Divya Bhardwaj**: Investigation, resources, formal analysis, methodology, writing‐original draft, review, editing. **Karina Quiaoit**: Investigation, resources, formal analysis, methodology, writing‐original draft, review, editing. **Murtuza Saifuddin:** Investigation, resources, formal analysis, methodology, writing‐original draft, review, editing. **Irene Karam:** Investigation, resources, formal analysis, methodology, writing‐original draft, review, editing. **Ian Poon:** Investigation, resources, formal analysis, methodology, writing‐original draft, review, editing. **Zain Husain:** Investigation, resources, formal analysis, methodology, writing‐original draft, review, editing. **William T. Tran**: Investigation, resources, formal analysis, methodology, writing‐original draft, review, editing. **Lakshmanan Sannachi**: Investigation, resources, formal analysis, methodology, writing‐original draft, review, editing. **Gregory J. Czarnota:** Study concept, **i**nvestigation, funding, project administration, resources, formal analysis, methodology, writing‐original draft, review, editing.

## Funding information

Terry Fox Foundation Program Project Grant from the Hecht Foundation (grant number 1083).

## Supporting information

Fig S1

Table S1

## Data Availability

Anonymized data will be made available on request in accordance with institutional policies.
