# Supplementary figures and images for: Quantitative ultrasound radiomics in predicting recurrence for patients with node‐positive head‐neck squamous cell carcinoma treated with radical radiotherapy
Source: Cancer Med. 2020 Dec 13;10(8):2579–89. doi: 10.1002/cam4.3634 (PMC8026932; doi:10.1002/cam4.3634)

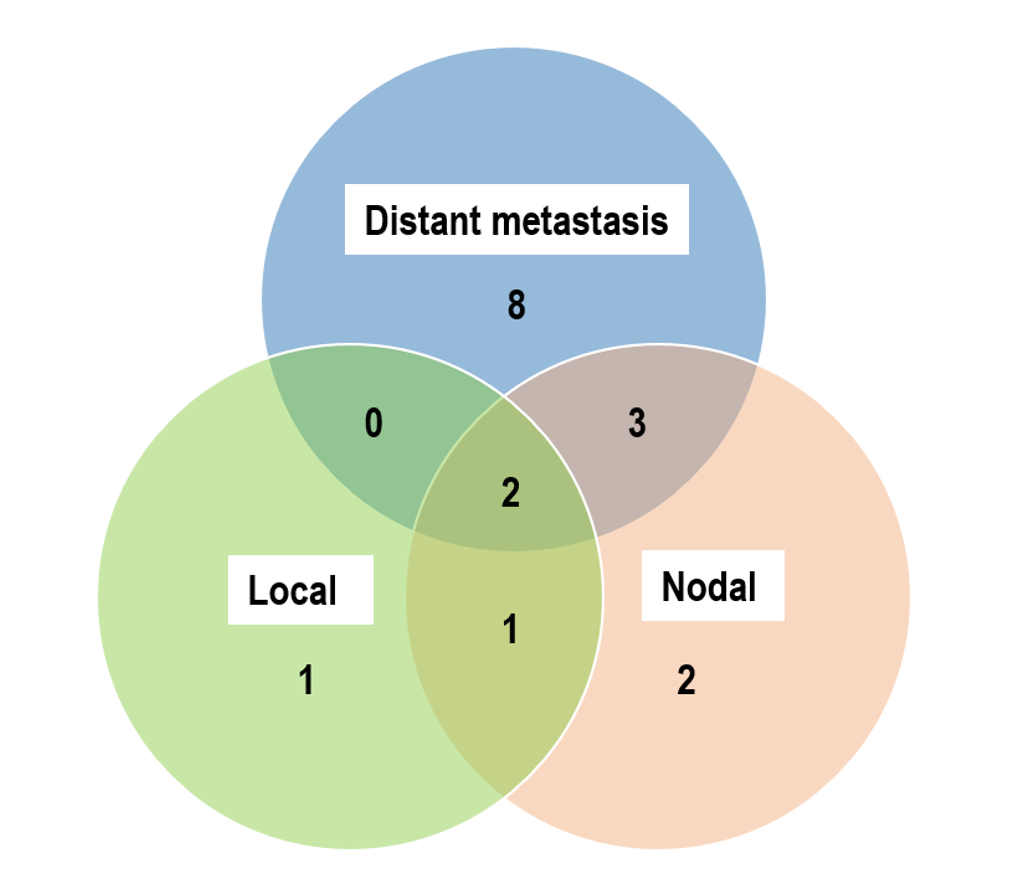

Supplement: Supplementary file 1 — Fig S1 [file CAM4-10-2579-s001.tif]
