# Supplementary material for: Quantitative ultrasound radiomics in predicting recurrence for patients with node‐positive head‐neck squamous cell carcinoma treated with radical radiotherapy
Source: Cancer Med. 2020 Dec 13;10(8):2579–89. doi: 10.1002/cam4.3634 (PMC8026932; doi:10.1002/cam4.3634)
Supplement: Supplementary file 2 — Table S1 [file CAM4-10-2579-s002.docx]

**Supplementary Table 1: Features with statistically significant difference between the two groups: recurrence (R) vs no recurrence (NR)**

| **Parameter** | **p-value** | **Mean Value (NR)** | **Mean Value (R)** |
| --- | --- | --- | --- |
| **SAS-CON** | 0.049 | 9.850±1.693 | 14.920±3.041 |
| **ASD-ENE** | 0.026 | 0.091±0.012 | 0.158±0.030 |
